# Properties of Artificial Phospholipid Membranes Containing Lauryl Gallate or Cholesterol

**DOI:** 10.1007/s00232-018-0025-z

**Published:** 2018-03-07

**Authors:** Małgorzata Jurak, Robert Mroczka, Rafał Łopucki

**Affiliations:** 1 Department of Physical Chemistry - Interfacial Phenomena, Faculty of Chemistry, Maria Curie-Skłodowska University, Maria Curie-Skłodowska Sq. 3, 20-031 Lublin, Poland; 2 Laboratory of X-ray Optics, Center for Interdisciplinary Research, The John Paul II Catholic University of Lublin, Konstantynów 1, 20-708 Lublin, Poland

**Keywords:** Lauryl gallate, Phospholipids, Cholesterol, Langmuir monolayers, Brewster angle microscopy

## Abstract

Lauryl gallate (LG) is an antioxidant agent. However, it exhibits poor solubility in water. Its interactions with the membrane result in structure evolution thus affecting the membrane functionality. In this paper the Brewster angle microscope coupled with the Langmuir trough was applied to determine the morphology, phase behaviour, thickness and miscibility of ternary Langmuir monolayers with equal mole fractions of 1,2-dipalmitoyl-*sn*-glycero-3-phosphocholine (DPPC); 1,2-dioleoyl-*sn*-glycero-3-phosphocholine (DOPC) and an increasing mole fraction of LG. The results were discussed as regards analogous systems where cholesterol (Chol) was the third component. Moreover, the phosphatidylcholine–lauryl gallate (PC–LG) interactions were monitored by the attenuated total reflectance Fourier transform infrared spectroscopy and time-of-flight secondary ion mass spectrometry. Besides lipid composition, the addition of LG was found to be a significant factor to modulate the model membrane properties. The LG molecules adjust themselves to the PC monolayer structure. The hydrophobic fragment is dipped into the membrane interior while the hydroxyl groups of phenolic gallate moiety associate with the polar groups of PC mainly through hydrogen bonding inducing the compacting effect. LG is found to be deeply submerged within DOPC, closer to the double bonds, and its insertion practically does not affect the DPPC/DOPC membrane fluidity. This is crucial for getting more profound insight into the role of LG in stabilizing the non-raft domains, mostly exposed to oxidation in which LG can co-localize and serve its antioxidant function.

## Introduction

Domain formation in the model membranes has been intensively studied since the hypothesis of lipid rafts was put forward (Simons and Ikonen 1997). Rafts are defined as nanoscale regions of biological membranes enriched in cholesterol, saturated long-chained lipids and particular proteins. They take part in important cellular processes and signalling pathways as well as pathogens can use these specific regions to infect a cell (Gatfield and Pieters 2000). Model membranes, such as lipid Langmuir monolayers at the air–water interface, offer the possibility of understanding how phase separation and domain formation can be regulated by the lipid components (Veatch and Keller 2005). A number of lipid mixtures was used to mimic the biophysical properties of rafts in membrane (Veatch and Keller 2002). The local lipid composition affects the size of lipid rafts. The addition of subsequent component can initiate the formation of larger membrane domains detectable by optical microscopy (Eeman and Deleu 2010). Introduction of cholesterol modifies the membrane physical stability by altering its fluidity, permeability and dielectric properties (McMullen et al. 2004). The separation of ordered areas from the disordered (fluid) regions is a natural consequence of specific intermolecular interactions and lattice deformation. Formation of such domains can be modulated depending on the lipid composition. Creation of lipid domains is a key process by which biomembranes function (Eze 1991). Depending on the type of lipids, the ratio size of the domains can vary. The size, structure and stability of the domains are determined by the variety of intermolecular forces occurring between particular components in the layer. These are mainly the van der Waals forces, steric, dipole–dipole, electrostatic interactions or hydrogen bond networks. Moreover, the domain structure can be changed as a result of preferential hydrolysis of the phospholipids (PL) by interfacially activated enzymes (Simonsen 2008) or can be affected by agents such as solvents, detergents, nanoparticles or drugs (Berquand et al. 2004; Fa et al. 2007; El Kirat et al. 2010). Interactions of such substances with biological membranes can inform about their activity and/or toxicity.

In contrast to rafts the membranes contain laterally segregated regions (domains) enriched in unsaturated PL susceptible to oxidative attack. Oxidation of unsaturated fatty acids in biological membranes lowers the membrane fluidity, damages its structure and functionality and finally causes undesirable lesions and diseases (Yagi 1987). To reduce the risk of pathological changes many efforts are put into developing safe and effective antioxidants. Lauryl gallate (LG), the *n*-alkyl ester of gallic acid, emerged as a good candidate to reduce cell damage induced by hydroxyradicals and hydrogen peroxides (Kubo et al. 2002, 2010). LG is widely used in food industry as well as in pharmaceutical and cosmetic manufacturing. By scavenging of the hydroxyl radicals, alkyl gallate is found to be an effective agent to prevent the dermal fibroblast cells from damage (Masaki et al. 1997). Moreover, it protects mitochondrial functions and human red blood cells from oxidative stresses (Kubo et al. 2002). LG has already been identified as a membrane binding agent (Takai et al. 2011). As a hydrophobic antioxidant it is likely to penetrate into the interior of membranes of cells and organelles and, similarly to α-tocopherol (Atkinson et al. 2010), it concentrates in the regions where the oxidation is supposed to occur. One can hypothesize that contrary to cholesterol, LG preferably incorporates into the unsaturated PL-rich domains thus producing local concentration amplification to optimize the protection of membrane areas most exposed to oxidation. Thus studies with a broad range of LG mole fractions in the PL membranes are an important issue. On the other hand, similarly to the role of cholesterol in rafts, LG can stabilize non-raft domains enriched in unsaturated PL as it was hypothesized for the other antioxidant α-tocopherol (Atkinson et al. 2010). This antioxidant activity can be correlated with the direct interactions of gallate with the components of biological membranes in which lateral phase separation occurs. However, the influence of LG on stability and fluidity of the membrane, especially the heterogeneous ones, is poorly understood. So far little has been known about LG effect on the phase separation of the membrane components and the size of the domains. The Langmuir monolayer studies of the ternary systems with LG have not been carried out yet.

The aim of this work was to investigate the phase behaviour, morphology and stability of model phospholipid membranes containing cholesterol or antioxidant LG. From the wide spectrum of gallates only LG seemed to optimize high antioxidant activity with sufficient hydrophobicity for the Langmuir monolayer formation. The effect of LG on the binary DPPC/DOPC monolayers was compared to that of cholesterol, which unlike LG is a structural component of biological membranes. The ordering of PL induced by Chol is driven by steric interactions (Marquardt et al. 2016). The LG molecules seem to adjust themselves to the PC monolayer structure by possible multiple hydrogen bond formation with the PC headgroups and the hydrophobic interactions of dodecyl tail dipped into the membrane interior. Such localization could affect the packing of membrane components and determine the protection of membrane against oxidation.

For these purposes, the ternary DPPC/DOPC/Chol (or LG) Langmuir films on the water subphase were investigated using the Brewster angle microscope (BAM) coupled with the Langmuir trough. The kind and magnitude of interactions between molecules in the ternary systems were estimated based on the experimentally determined surface pressure–area per molecule (*π*–*A*) isotherms (excess area, excess Gibbs energy, total Gibbs energy of mixing). Moreover, the PC–LG interactions in the hydrated multibilayers were monitored by the attenuated total reflectance Fourier transform infrared (ATR-FTIR) spectroscopy. The studies were supplemented with the time-of-flight secondary ion mass spectrometry (TOF-SIMS) analysis which identified chemically the lateral distribution of components in the PC/LG monolayers. Formation of intermolecular hydrogen bonds between the galloyl moiety and the head groups of PC was examined based on the signal intensity of characteristic ions.

The experiments performed on the pure water subphase were the basic stage in determining the interactions of LG with a model membrane, necessary for further studies. The next step was deposition of the floating monolayers onto the solid support. To reduce the risk of possible changes in the native film structure, transfer was also made from the pure water subphase.

The application of the PL and LG mixtures of defined stoichiometry, in the full range of molar ratios, ensures control of the membrane composition, its physical state and thickness. This is the basic research enabling better understanding of the nature of the interactions between components in the ternary systems that are crucial for stabilizing the non-raft domains most exposed to oxidation. It is believed that studies of miscibility of LG with PC phases at the air/water interface can be helpful for getting better insight into the mode of LG action on the membrane level as well as development of a new generation of antioxidants which could be incorporated into lipocarriers used in pharmaceutical, food and cosmetic formulations.

## Experimental

### Materials

PL: 1,2-dipalmitoyl-*sn*-glycero-3-phosphocholine (DPPC) and 1,2-dioleoyl-*sn*-glycero-3-phosphocholine (DOPC), cholesterol (Chol) and LG of purity > 99% were provided by Sigma and used without purification. Chloroform (p.a.) and ethanol (96%, p.a.) (Avantor Performance Materials Poland S.A.) were solvents for compound dissolution. Redistilled water was purified by the Milli-Q system to obtain resistivity of 18.2 MΩcm. Such water was used as a subphase for the Langmuir monolayer formation.

### Methods

#### Langmuir Monolayer Formation

The one-component (DPPC, DOPC, Chol, LG), binary (DPPC/DOPC 1:1) and ternary (DPPC/DOPC/Chol and DPPC/DOPC/LG) Langmuir monolayers were formed on the water subphase using a computer-controlled KSV standard trough (KSV Instruments Lt., Finland). First the compounds were dissolved in chloroform to obtain the total concentration of 1 mg/mL. Then the binary mixture of DPPC/DOPC (*x* = 0.5) and the ternary mixtures of the constant DPPC and DOPC ratio (*x* = 0.5) with varying Chol or LG mole fraction (*x* = 0.25; 0.5; 0.75) were prepared from the respective stock solutions. The droplets of the solution were placed on the subphase surface top by means of a Hamilton microsyringe. After chloroform evaporation, the surface pressure–area per molecule (*π*–*A*) isotherms were recorded during symmetrical monolayer compression with the rate of 10 mm/min. The solubility of LG in water was estimated for 0.035 mg/mL, i.e. 1.034 × 10^−4^ M (http://www.hmdb.ca/metabolites/HMDB38720). It was sufficiently low to form the Langmuir monolayers on water in the wide range of compression rates (from 5 to 100 mm/min) and its monolayers were stable in time even at a high surface pressure. The temperature of 20 °C was maintained owing to the water system circulation (thermostat Alpha RA 8, Lauda). The surface pressure was measured by the Wilhelmy plate method. Simultaneously, the morphology of the floating monolayers was monitored by the BAM coupled with the trough. The Langmuir trough and BAM were placed in the laser safety cabinet and isolated from vibrations coming from the surroundings by the active vibration isolation system.

#### Brewster Angle Microscopy (BAM) Observation and Relative Thickness Evaluation

A computer-controlled nanofilm_ultrabam (Accurion GmbH, Germany) was used for direct visualization of substructures with a long range orientational order and for evaluation of layer thickness at the air–water interface. The light source of the BAM was a solid-state 50 mW laser-emitting 658 nm wavelength light. The laser beam accounts for purely *p*-polarized light, vertically linearly polarized or light polarized in the plane of incidence. Before getting to the monolayer/water surface, the light went through a polarizer to reduce the polarization ratio (non-polarized light/total light) to 10^−8^. As *p*-polarized light illuminated the pure water surface at the Brewster angle of about 53.2°, practically no intensity reflection was observed (dark background without contrast). When the monolayer with the refractive index different from that of water (i.e. 1.33) was spread onto the subphase, reflection occurred. The reflected light passed through the objective lens into the analyzer, and finally to the CCD camera (1392 × 1040 pixels, max 40 fps). These elements were positioned in the reflected beam path to acquire high quality contrast images of the lateral morphology. Imaging of 720 µm × 400 µm areas was performed with the lateral resolution of 2 µm. The black glass plate was placed under the water subphase on the bottom of the trough to prevent diffraction of the laser beam and to minimize light scattering.

To determine the relative film thickness in each case first the camera calibration was made to determine the relationship between the grey level and relative reflectivity (Rodriguez Patino et al. 1999). During calibration the plot (parabola) of grey level as a function of the angle of incidence was obtained. The minimum of the parabolic fit was the angle of incidence with the lowest reflectivity valid under the current environmental conditions. Owing to the calibration factor it was possible to convert greyscale information into reflectivity. Then the single-layer optical model (Eq. 1) was applied to convert the reflectivity *R* into the film thickness *d* (Winsel et al. 2003).

1 $$R=\frac{{{I_{\text{r}}}}}{{{I_0}}}={\left( {\pi \frac{d}{\lambda }} \right)^2}\frac{{{{\left( {n_{1}^{2} - n_{2}^{2} - 1+\frac{{n_{2}^{2}}}{{n_{1}^{2}}}} \right)}^2}}}{{1+n_{2}^{2}}},$$

where *I*_0_ and *I*_r_ are the incident and the reflected intensity, respectively; *n*_1_ and *n*_2_ denote the refractive indices of the film and the subphase, respectively; *λ* is the wavelength of the incident light.

#### ATR-FTIR Measurements

The multibilayers built of DPPC or DOPC and LG at different mole fractions (0.25 and 0.5) were prepared on the ZnSe crystal by spreading of the chloroform solutions. Knowing the geometric surface area of the ZnSe crystal (3.5 × 10^−4^ m^2^) and the mean molecular areas of components in the monolayers (*A*_12_), as determined from the *π*–*A* isotherm at 35 mN/m, first the quantity of compounds needed to cover the support with one statistical monolayer was calculated. Then the concentration and volume of the chloroform solutions were chosen so that after evaporation of the solvent, the 40 statistical bilayers were formed in each case. Such number of bilayers was found to be sufficient for obtaining good quality spectra. Before the solution pouring, the ZnSe crystal plate was cleaned with ultra pure ethanol. After the solvent evaporation the supported layers were transferred to a vacuum (117 mbar) for 30 min to remove the possible chloroform residuals, and then placed into the closed chamber with a relative humidity 80% for 30 min in order to hydrate the dried multibilayers.

Infrared absorption spectra were obtained with a Nicolet 8700A Thermo Scientific spectrometer. The attenuated total reflection (ATR) configuration was used with the Varimax HATR ZnSe crystal plate (45° cut). Typically 256 Fourier-transformed and averaged scans were collected for each measurement. Absorption spectra at a resolution of 4 cm^−1^ were recorded in the region between 4000 and 650 cm^−1^ using a clean crystal as the background. All the experiments were performed at room temperature using automatic atmospheric correction. Spectral analysis was conducted with the Omnic software from Thermo Scientific (USA).

#### TOF-SIMS Measurements

The Langmuir monolayers of DPPC, DOPC, LG and mixed PC/LG (*x*_LG_ = 0.25) were transferred at 35 mN/m with the rate of 5 mm/min from the water subphase onto the mica plates using the Langmuir–Blodgett trough (KSV, Finland). The samples were dried under vacuum (117 mbar) overnight. Mass spectrometric measurements were made using a TOF-SIMS 5 device (ION-TOF GmbH, Germany). The primary ion source of Bi_3_^+^ was used at 25 keV. The scanning area of secondary ions was 100 µm × 100 µm with 256 × 256 pixels. All measurements were performed in the static mode (dose no larger than 10^13^ ions/cm^2^). Two different places of each sample were analysed. No statistical difference was found between them. The post processing data analysis was conducted using the SurfaceLab 6.7 software (ION-TOF). For all samples charge effects were compensated using a flood gun adjusted individually for each sample area. Only positive spectra were recorded and calibrated using the positions of CH_3_^+^, C_4_H_7_^+^, C_5_H_9_^+^ peaks. The mass peaks with m/z of 27, 29, 39 and 58 were removed and then the mass spectra were compared after normalization of the intensity, proportionally to the total intensity recorded for each spectrum.

## Results

### *π*–*A* Isotherms

**Fig. 1:**
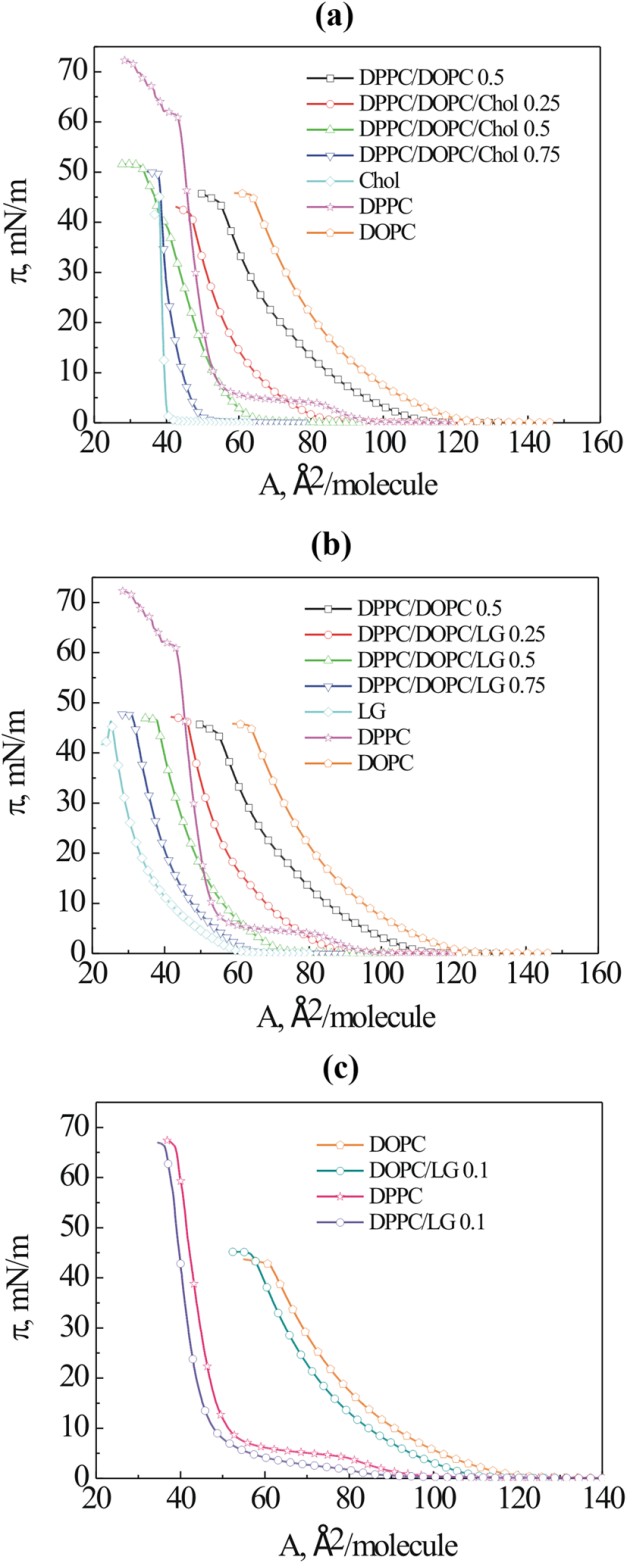
Surface pressure–area (*π*–*A*) isotherms of the pure DPPC, DOPC, Chol, LG, binary DPPC/DOPC, ternary DPPC/DOPC/Chol (**a**) and DPPC/DOPC/LG (**b**) monolayers registered on the water subphase at 20 °C. The *π*–*A* isotherms of the mixed DPPC/LG or DOPC/LG (*x*_LG_ = 0.1) monolayers, in which it was used the same amount of PC as in the pure DPPC or DOPC monolayers, are also presented (**c**). (Color figure online)

The effect of Chol or LG on the interactions and phase behaviour of lipidic constituents of biological membranes: DPPC, and DOPC, was examined in the ternary DPPC/DOPC/Chol (or LG) mixtures by monitoring differences in the shape of the compression isotherms at the air–water interface. Figure 1 presents the isotherms of pure DPPC, DOPC, Chol and LG, binary DPPC/DOPC (1:1) and ternary monolayers. The characteristic first order phase transition LE–LC occurs only for DPPC. The DOPC monolayer exhibits a single homogeneous LE phase up to the collapse. The *π*–*A* isotherm of DPPC/DOPC monolayer does not show any inflections which would indicate the LE–LC phase transition. For all studied systems the isotherms of ternary monolayers are between those of DPPC/DOPC and one-component Chol or LG monolayers. With the increased Chol or LG mole fraction in the DPPC/DOPC monolayers, the isotherms shift toward smaller molecular areas due to the smaller area occupied by Chol or LG molecule than that of DPPC/DOPC and/or the mutual interactions. For the mixed monolayers at the air–water interface the surface pressure of collapse depends on composition. Generally, if components are miscible, only one collapse point exists which varies with the mixed film composition. In contrast, when both components are immiscible, two collapse states occur which can be attributed to the collapse surface pressures for the individual pure components. Then the collapse pressure is constant regardless of the composition of the mixed films (Gaines 1966). Therefore the component collapsing at lower surface pressure should be expelled from such mixed monolayer at the surface pressure of collapse of the pure component independently of its content in the initially spread mixed film. However, this does not take place for the investigated systems. The pure LG monolayer collapses at high surface pressure which is close to that of DOPC and DPPC/DOPC monolayer (Fig. 1b).

To prove that LG is not ‘squeezed out’ of the PC monolayer, the isotherm of pure DOPC monolayer was recorded and then taking the same amount of DOPC the mixed DOPC/LG film containing 10 mol% LG was obtained. The measurements were repeated three times. The same experiment was conducted for the systems with DPPC instead of DOPC. The results are presented in Fig. 1c. In both cases the presence of LG causes decrease of molecular area. There are not visible any characteristic kinks on the *π*–*A* isotherms which would suggest LG leaving the monolayer for the bulk subphase, or forming a multilayered film. These findings allow to confirm that the LG molecules remain in the mixed monolayer and associate with PC in such a way that changes the polar head configuration yielding smaller areas per molecule.

Moreover, as reported previously for the DOPC/LG, POPC/LG and DPPC/LG binary systems in the full range of the LG mole fraction, no LG squeezing out of the PC monolayers was found (Jurak and Miñones 2016). There was no evidence of phase separation on the BAM images for both POPC/LG and DOPC/LG mixtures until the collapse took place. The authors concluded that the components of the mixed monolayers were fully miscible. The existence of a single collapse pointed out that all components were kept in the monolayer. The above statement concerns also the ternary systems (Fig. 1a, b). Here the first collapse pressure alters with the monolayer composition indicating the non-ideal behaviour. These changes can be clearly seen for DPPC/DOPC/Chol. In the case of DPPC/DOPC/LG they are significantly smaller therefore the conclusion regarding miscibility based on the collapse pressure analysis is not so evident and further analysis of miscibility was conducted (paragraph Interactions).

### Morphology

**Fig. 2:**
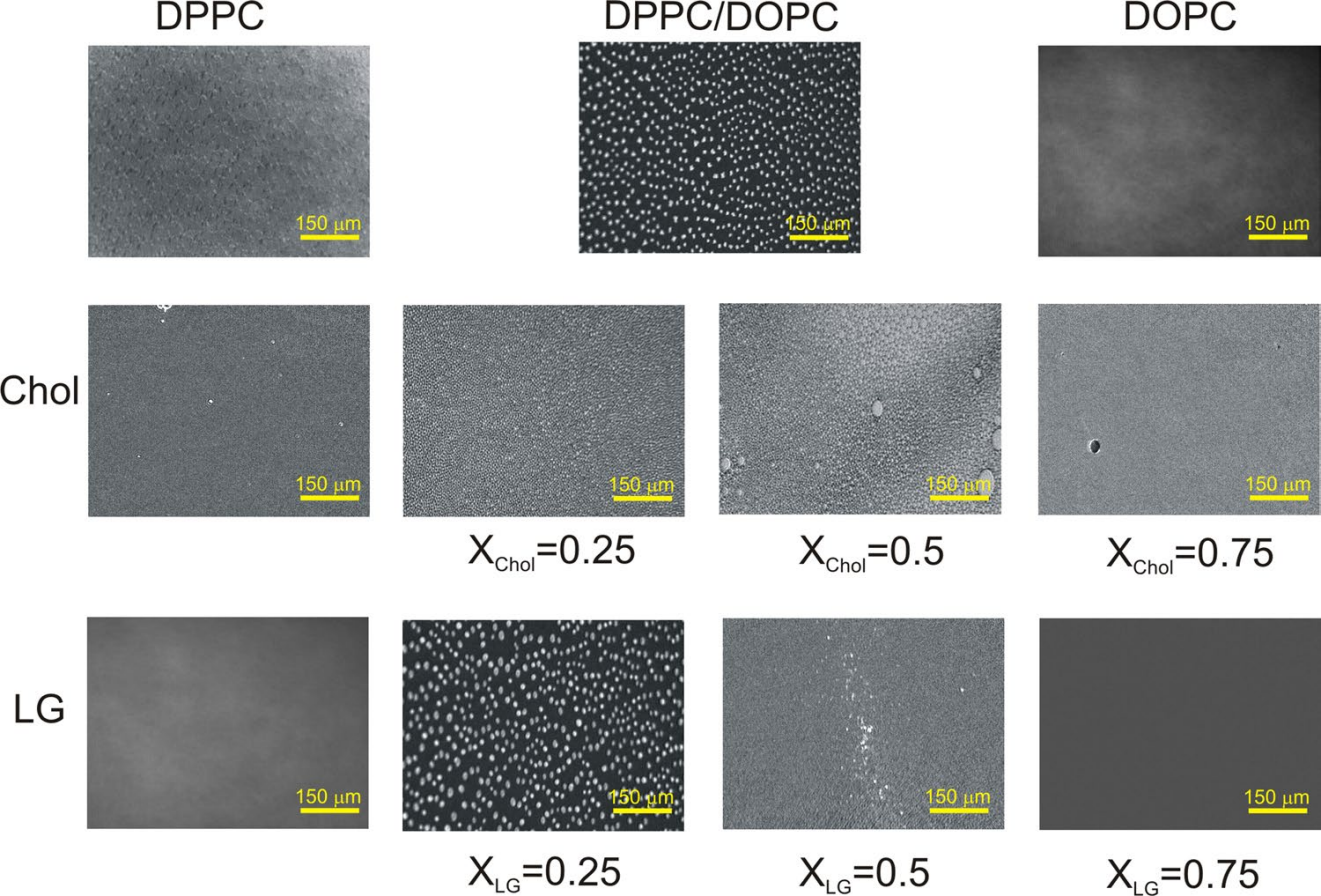
BAM images of the pure DPPC, DOPC, Chol, LG, binary DPPC/DOPC, ternary DPPC/DOPC/Chol and DPPC/DOPC/LG monolayers imaged on the water subphase at 20 °C. The field of view is 720 µm × 400 µm

Morphology of floating monolayers was observed simultaneously as the *π*–*A* isotherms were recorded. However, the images obtained only at 35 mN/m are presented in Fig. 2. Such value of surface pressure corresponds to the lateral pressure value of biological membranes (Marsh 1996). Accordingly, the phase behaviour, miscibility and stability were also analysed at that surface pressure.

**Fig. 3:**
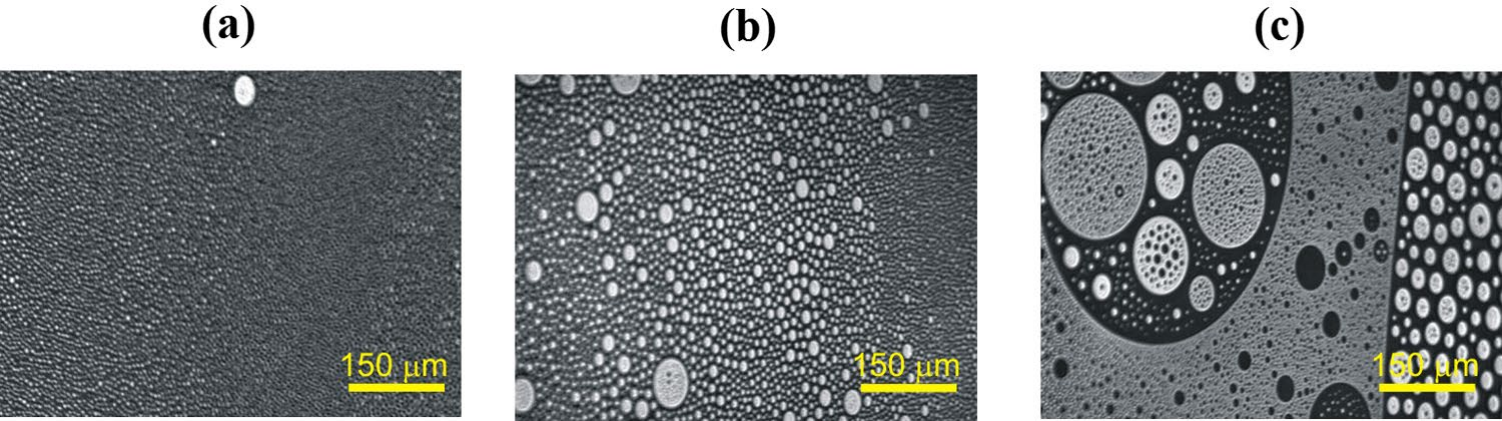
BAM images of the ternary DPPC/DOPC/Chol monolayers with different Chol molar ratios, i.e. 0.25 (**a**), 0.5 (**b**) and 0.75 (**c**) imaged on the water subphase at 0.5 mN/m and 20 °C. The field of view is 720 µm × 400 µm

Both DPPC and DOPC form homogeneous monolayers at 35 mN/m but that of DPPC is tightly packed (condensed) while that of DOPC is loosely packed (expanded) (Fig. 2). In the mixed DPPC/DOPC monolayer the partial miscibility takes place leading to coexistence of the condensed domains enriched in DPPC and fluid matrix enriched in DOPC. In the BAM images there are present small irregularly shaped condensed domains in the mixed monolayer whose sizes and total amount are smaller than those of DPPC at the LE–LC phase transition (not shown here). Moreover, they appear at the higher surface pressure (21–23 mN/m) than that at which the LE–LC phase transition takes place. They grow during compression and reach the maximal size at 35–38 mN/m. With the further increase of surface pressure the domains become smaller. This observation is consistent with that previously reported by Nag and Keogh (1993). In the presence of Chol even at the surface pressure < 0.5 mN/m there appear some circular domains exhibiting different morphology depending on the Chol mole fraction (Fig. 3). At low Chol content (*x*_Chol_ = 0.25) there is observed the liquid–liquid phase coexistence (region of coexisting liquid phases) with one phase rich in the unsaturated DOPC and the other in the saturated DPPC and cholesterol which can be seen as dark and bright regions, respectively. Hence the bright regions seem to be equivalent to the liquid-ordered state (Veatch and Keller 2003). During compression small circular domains merge partially while deforming when domains collide to form a packed structure at 35 mN/m (Fig. 2). Even at *x*_Chol_ = 0.5 two liquid phases still coexisting as micron-sized liquid domains are observed and their heterogeneity of size is larger than that at *x*_Chol_ = 0.25. At a high mole fraction (*x*_Chol_ = 0.75) one uniform liquid phase is observed at first as a result of coalescence of domains into larger ones, and finally at ca. 30 mN/m into one homogeneous phase. Domains coalesce close to a critical point due to a small difference in the dipole density between phases (Keller and McConnell 1999). The observed coexisting liquid phases are in agreement with the phase diagrams of the ternary DPPC/DOPC/Chol system reported in the literature (Veatch and Keller 2002, 2003; Veatch et al. 2004; Almeida 2009).

The addition of LG into the DPPC/DOPC binary system causes spontaneous formation of liquid domains from the uniform monolayers at different surface pressures depending on the LG content, i.e. at *x* = 0.25–21 mN/m; at *x* = 0.5–30 mN/m; at *x* = 0.75–38 mN/m. At low LG concentration *x*_LG_ = 0.25 and surface pressure, the area fraction of bright oval domains is small but the average size of the condensed domains increases with the increasing surface pressure up to 35 mN/m. Contrary to DPPC/DOPC, further compression does not lead to the change of domain sizes or shapes but it increases only their number per unity area. Hence the domains can be identified individually. At *x*_LG_ = 0.5 the domains are much smaller and rather sparsely spread. With the LG mole fraction increase up to *x* = 0.75 the ternary monolayer is homogeneous at 35 mN/m (Fig. 2). After collapse some additional aggregates appear.

### Membrane Packing

In order to verify and compare the physical state of studied films as well as to obtain information about the molecular ordering in the monolayers, the compression modulus $C_{{\text{s}}}^{{ - 1}}$ was calculated for the films of known composition by the numerical calculation of the first derivative from the *π*–*A* isotherm data using Eq. 2:

2 $$C_{{\text{s}}}^{{ - 1}}= - A{\left( {\frac{{{\text{d}}\pi }}{{{\text{d}}A}}} \right)_{T,n}},$$

where *A* denotes the area per molecule at a given surface pressure *π*.

**Fig. 4:**
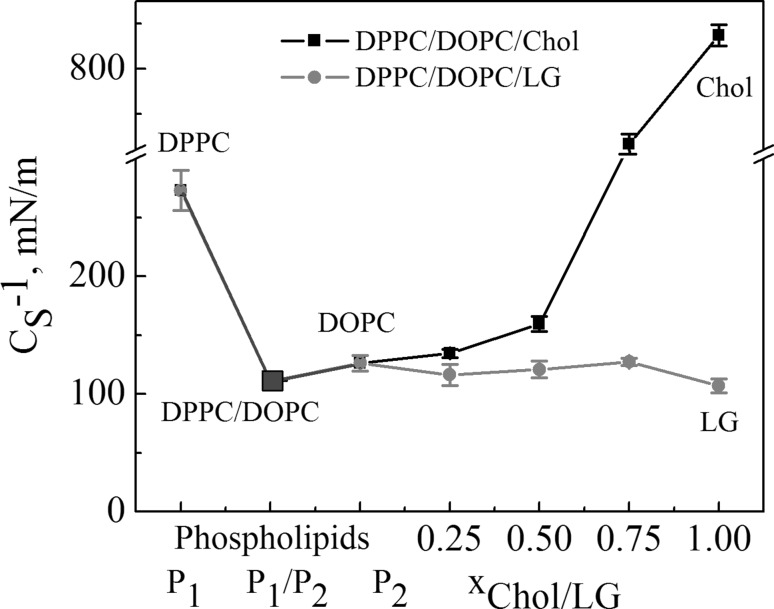
Compressibility modulus ($C_{{\text{s}}}^{{ - 1}}$) versus composition of the monolayers at 35 mN/m. (Color figure online)

The $C_{{\text{s}}}^{{ - 1}}$ values obtained at 35 mN/m are presented in Fig. 4. The compression modulus of DOPC indicates its liquid-expanded phase while that of DPPC is the liquid-condensed state. The addition of DOPC to DPPC yields an increase of monolayer fluidity and therefore the mixed DPPC/DOPC monolayer is characterized by the value of $C_{{\text{s}}}^{{ - 1}}$even lower than that of DOPC. On the other hand, the compression modulus of pure Chol is by about 840 mN/m higher than that of LG, and proves that Chol exists as a condensed state while LG as a liquid-expanded state. The presence of Chol in the DPPC/DOPC monolayer decreases membrane fluidity and therefore the gradual increase of the compression modulus values with the Chol mole fraction is observed while those of DPPC/DOPC/LG change with composition only slightly. At a low Chol (LG) content (*x* = 0.25) in DPPC/DOPC the difference in $C_{{\text{s}}}^{{ - 1}}$ between both ternary monolayers is only 18 mN/m while at *x* = 0.75 it is as large as 337 mN/m. The drastic increase in the degree of the monolayer condensation, i.e. packing of the lipid molecules, makes it more ordered.

Additionally, the geometric packing (arrangement) of molecules was described in terms of the molecular shape of the constituents. It was predicted based on the critical packing parameter *s* (Israelachvili 2011). Its value determines the intrinsic (or spontaneous) curvature in the membrane, thereby the type of aggregate formed. Assuming that the polar headgroup surface area of LG *a* is estimated to be 87 Å^2^ (http://www.hmdb.ca/metabolites/HMDB38720), the volume of hydrocarbon chain *V* = 350.2 Å^3^, the length of hydrocarbon chain *l*_C_ = 16.68 Å, the critical packing parameter *s* was calculated according to Eq. 3 (Israelachvili 2011):

3 $$S=\frac{V}{{{l_{\text{C}}}a}}$$

The evaluated *s* parameter for LG is 0.24 which is lower than 1/3 suggesting a cone shape of molecule (Israelachvili 2011). This could induce a positive curvature stress in membranes. The corresponding critical *s* parameters for DPPC, DOPC and Chol are 0.57, 0.61 and 1.22, respectively, which ensures the truncated cone of PC and inverted truncated cone of Chol (Israelachvili 2011; Wnętrzak et al. 2013). The match of the molecular geometry of interacting LG or Chol components can be responsible for their affinity for the membrane PL. The combination of inverted truncated cone shape of Chol with the truncated cone shape of PC molecules provides a more favourable packing leading to the increased compression modulus. On the other hand, conically shaped LG seems to be less geometrically complementary to PC, i.e. the shape matching is not as optimal as for Chol. This can prevent formation of a tightly packed structure of monolayer. However, the critical *s* parameter does not include the specific interactions between the molecules which can dominate under the shape matching of the interacting molecules. As reported previously, Chol has an increased affinity for the membranes composed of DPPC compared to those of DOPC (Jurak 2013) while LG exhibits better affinity for the unsaturated PC (Jurak and Miñones 2016). LG practically does not change the monolayer LE phase state of DOPC irrespective of its mole fraction (Jurak and Miñones 2016), and it is more stable in the liquid phase of DPPC/DOPC than in the solid phase (Takai et al. 2011). Therefore similarity in the phase state of the LG and DOPC or DPPC/DOPC monolayers allows LG to accommodate within the membrane structure keeping its fluidity.

### Thickness

**Fig. 5:**
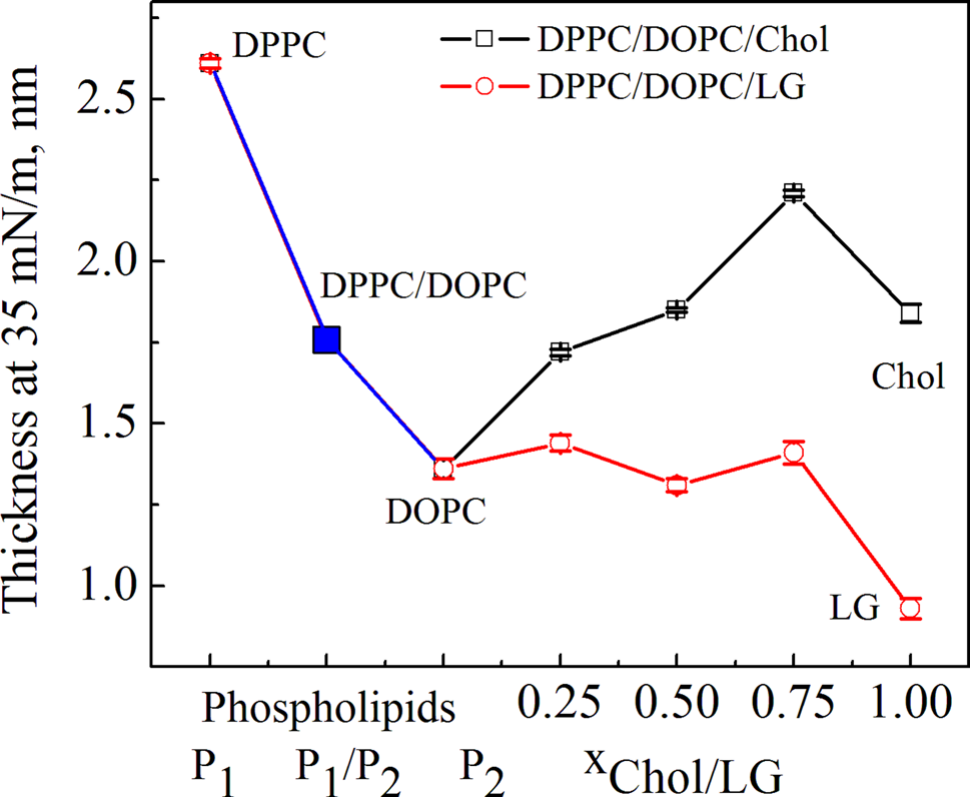
Thickness (*d*) versus composition of the monolayers at 35 mN/m. (Color figure online)

Although the monolayer of Chol is the stiffest, the greater thickness is obtained for the DPPC monolayer, which is 2.61 nm (Fig. 5). The DOPC molecules having two C18 unsaturated hydrocarbon chains give thinner monolayers (1.36 nm) than that of DPPC (C16) because of *cis*-double bonds preventing from straightening of the molecules. Intermediate thickness was determined for the DPPC/DOPC membrane (1.76 nm) for which coexistence of condensed DPPC-rich domains and a fluid DOPC-rich matrix can be seen (Fig. 2). With the mole fraction of Chol increase in the DPPC/DOPC mixture, the membrane thickness increases up to 2.21 nm at *x* = 0.75. This value is higher than that of pure Chol (1.84 nm). The thickness values of DPPC/DOPC/LG membranes depend insignificantly on the composition and they are located between those of DPPC/DOPC and LG. The thickness of pure LG monolayer is as low as 0.93 nm. The difference in thickness of both types of membranes becomes bigger as the mole fraction of the third component increases. As reported earlier Chol exerts a condensing effect on DPPC and DOPC (Jurak 2013) causing the increase of tight packing and ordering of acyl chains. Moreover, Chol shows greater affinity for fully saturated DPPC, and hence preferential interactions with the saturated species induce formation of cholesterol enriched domains according to the raft hypothesis. On the other hand, LG can cause either a rigidifying or fluidizing effect on DPPC depending on its amount in the mixed monolayer, i.e. 25% or ≥ 50%, respectively (Jurak and Miñones 2016). The DPPC monolayer thickness reported in the literature is in the range of 2.4–3 nm (Peterson and Russel 1985; Charitat et al. 1999; Balashev et al. 2003; Kienle et al. 2014), while that of the Chol monolayer is 1.6 nm (Gupta and Suresh 2004).

### Interactions

Miscibility and interactions between molecules in the mixed monolayers were qualitatively analysed in accordance with the additivity rule (Gaines 1966). Therefore mean molecular areas in the mixed films (*A*_123_) at different values of the surface pressure were determined directly from the isotherms, and then compared with the values obtained for the ideal miscibility or complete immiscibility of molecules based on Eq. 4:

4 $$A_{{123}}^{{{\text{id}}}}={A_1}{x_1}+{A_2}{x_2}+{A_3}{x_3},$$

where *A*_1_, *A*_2_, *A*_3_ denote the mean molecular areas in the one-component monolayers of DPPC, DOPC and Chol (or LG), respectively at a given surface pressure; and *x*_1_, *x*_2_, *x*_3_ are the molar ratios of components 1, 2 and 3 in the mixed monolayer.

**Fig. 6:**
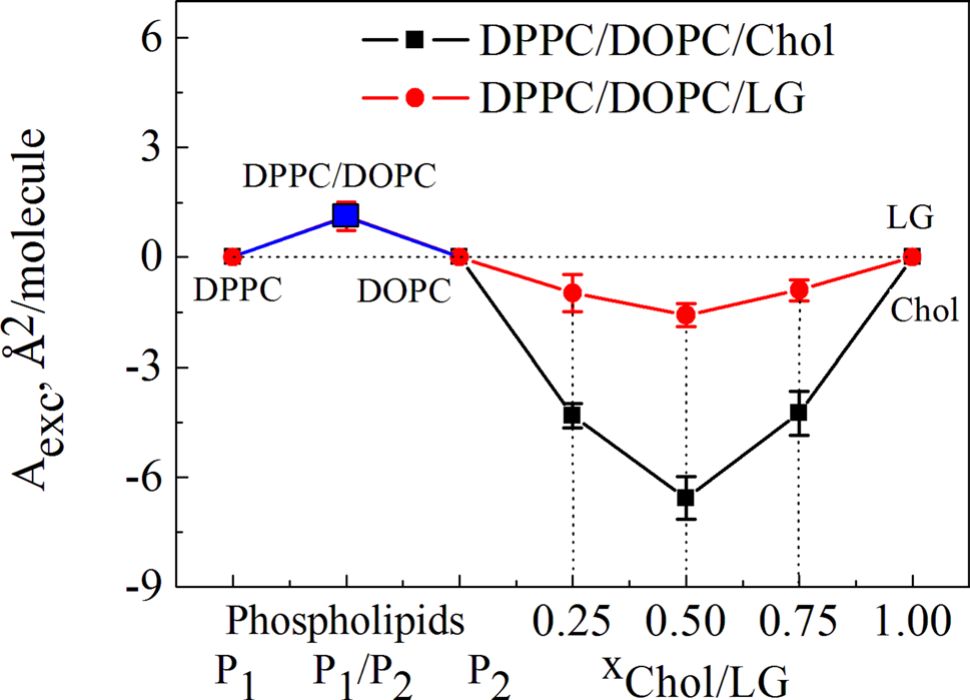
Excess area versus composition of the monolayers at 35 mN/m. (Color figure online)

Then the effect of Chol or LG on the DPPC/DOPC phospholipid films was estimated from the excess area per molecule values according to Eq. 5:

5 $${A_{{\text{exc}}}}={A_{123}} - A_{{123}}^{{{\text{id}}}},$$

where *A*_123_ is the mean molecular area in the ternary films. If *A*_exc_ is equal to 0, the dependence of *A*_123_ on the composition is linear, and the mixture behaves ideally indicating that the components are completely miscible or immiscible. When *A*_exc_ ≠ 0, i.e. any deviation from linearity of the *A*_123_ = *f* (*x*) function occurs, this indicates miscibility of the compounds due to the mutual interactions. The non-ideality of these mixed systems was expressed in the excess area (*A*_exc_)-composition plots shown in Fig. 6.

The excess area was calculated comparing the area per molecule in the mixed and pure monolayers at 35 mN/m (Eq. 5). The excess area per molecule as a function of Chol or LG molar fraction exhibits negative deviations from linearity which suggests that the interactions between heteromolecules in the ternary systems should be more attractive or less repulsive than those in the pure monolayers. For DPPC/DOPC/Chol, as the packing of molecules (the $C_{{\text{s}}}^{{ - 1}}$ values) and the monolayer thickness are higher than those of DPPC/DOPC/LG, the negative values of *A*_exc_ are lower (Fig. 6) due to the fact that more densely packed monolayers are formed.

To explore the magnitude of these interactions and to find out how different amounts of Chol or LG modulate their interactions with the DPPC and DOPC model membranes, the excess Gibbs energy of mixing (∆*G*_exc_) was determined at 35 mN/m from Eq. 6 (Gaines 1966):

6 $$\Delta {G_{{\text{exc}}}}=N\int\limits_{0}^{\pi } {{A_{{\text{exc}}}}d\pi },$$

where *N* denotes the Avogadro’s number, *A*_exc_ is the excess area, and *π* is the surface pressure which changes in the range of integration from 0 to 35 mN/m.

**Fig. 7:**
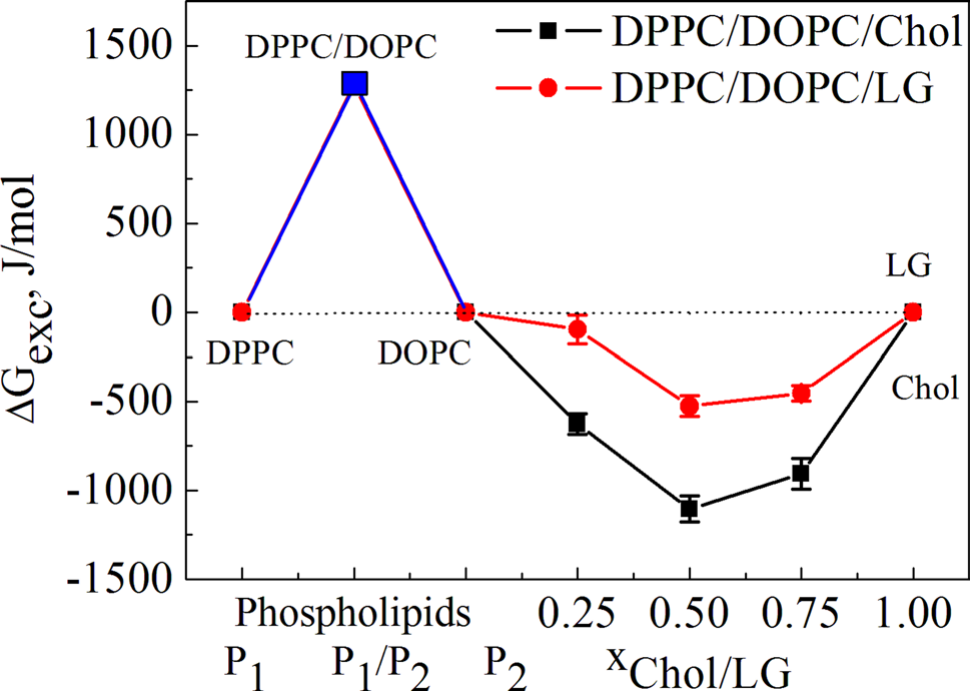
Excess Gibbs energy versus composition of the monolayers at 35 mN/m. (Color figure online)

The negative values of excess Gibbs energy (Fig. 7) confirm that the intermolecular interactions in the ternary films are more attractive than those in the ideal films. At a given molar ratio of the third component (LG or Chol), these intermolecular interactions are stronger for the systems with cholesterol for which the mixed monolayers are in a more condensed state (higher $C_{{\text{s}}}^{{ - 1}}$ values). However, the Δ*G*_exc_ minimum occurs at *x* = 0.5 for both systems (∆*G*_exc_ = − 1105 and − 526.5 J/mol, respectively). Although not corresponding to the more densely packed monolayers, at which $C_{{\text{s}}}^{{ - 1}}$ is maximal, it indicates the most favourable composition for the ternary systems. It is worth mentioning that Chol interacts preferably with saturated phospholipid DPPC while LG with unsaturated DOPC. It was also found that the strongest interactions between Chol and DPPC exist at *x*_Chol_ = 0.25 (∆*G*_exc_ = − 1660.9 J/mol) (Jurak 2013) whereas those between LG and DOPC occur at *x*_LG_ = 0.5 (∆*G*_exc_ = − 1424.2 J/mol) (Jurak and Miñones 2016).

Furthermore, in order to determine the thermodynamic stability of the mixed systems the total Gibbs energy of mixing was determined (Eq. 7):

7 $$\Delta {G_{\text{m}}}=\Delta {G_{{\text{exc}}}}+\Delta {G_{{\text{id}}}},$$

where the ideal Gibbs energy of mixing can be expressed as:

8 $$\Delta {G_{{\text{id}}}}=RT\left[ {{x_1}\ln {x_1}+{x_2}\ln {x_2}+{x_3}\ln {x_3}} \right],$$

where *R* is the gas constant and *T* is the temperature.

**Fig. 8:**
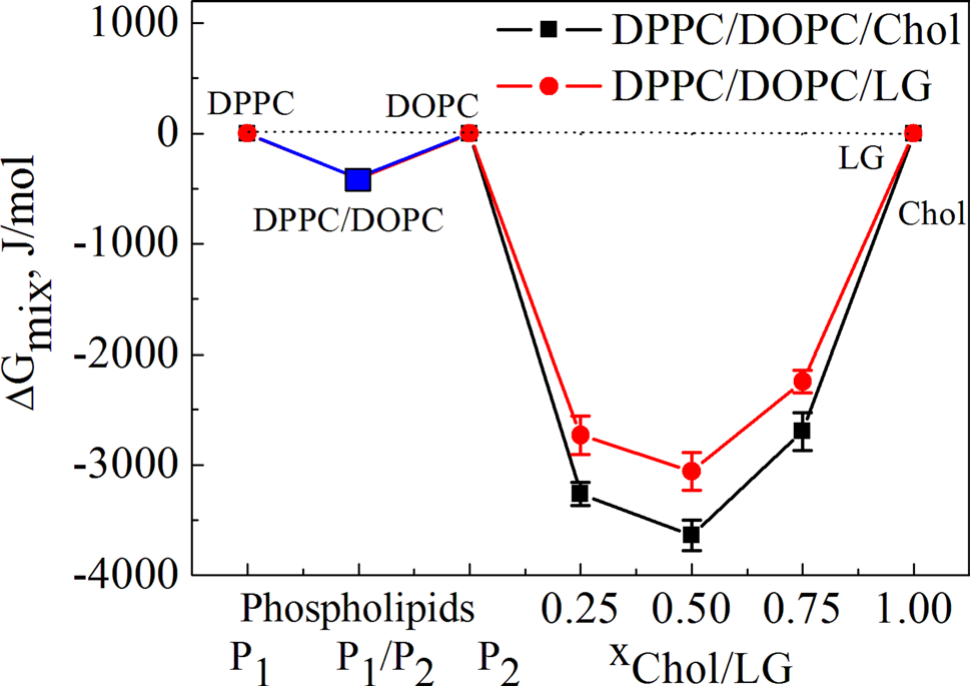
Total Gibbs energy of mixing versus composition of the monolayers at 35 mN/m. (Color figure online)

In all studied systems, the values of ∆*G*_mix_ are negative regardless of the monolayer composition (Fig. 8) confirming more thermodynamic stability of the ternary monolayers compared to the basic films. In spite of unfavourable DPPC–DOPC interactions the stability of ternary systems is similar (for DPPC/DOPC/LG) or higher (for DPPC/DOPC/Chol) in comparison to those of the binary systems PC/Chol (LG) which are miscible.

### ATR-FTIR Absorption Spectra Analysis

In order to understand better participation of functional groups in the interactions between DPPC or DOPC and LG the FTIR-ATR spectroscopy was applied for the hydrated PC/LG multibilayers formed on the ZnSe crystal. Such comparison was based on the monolayer–bilayer correspondence which implies that the properties of Langmuir monolayers at the surface pressure of 30–35 mN/m can be successfully correlated with those of the bilayer systems (Brockman 1999; Feng 1999).

**Fig. 9:**
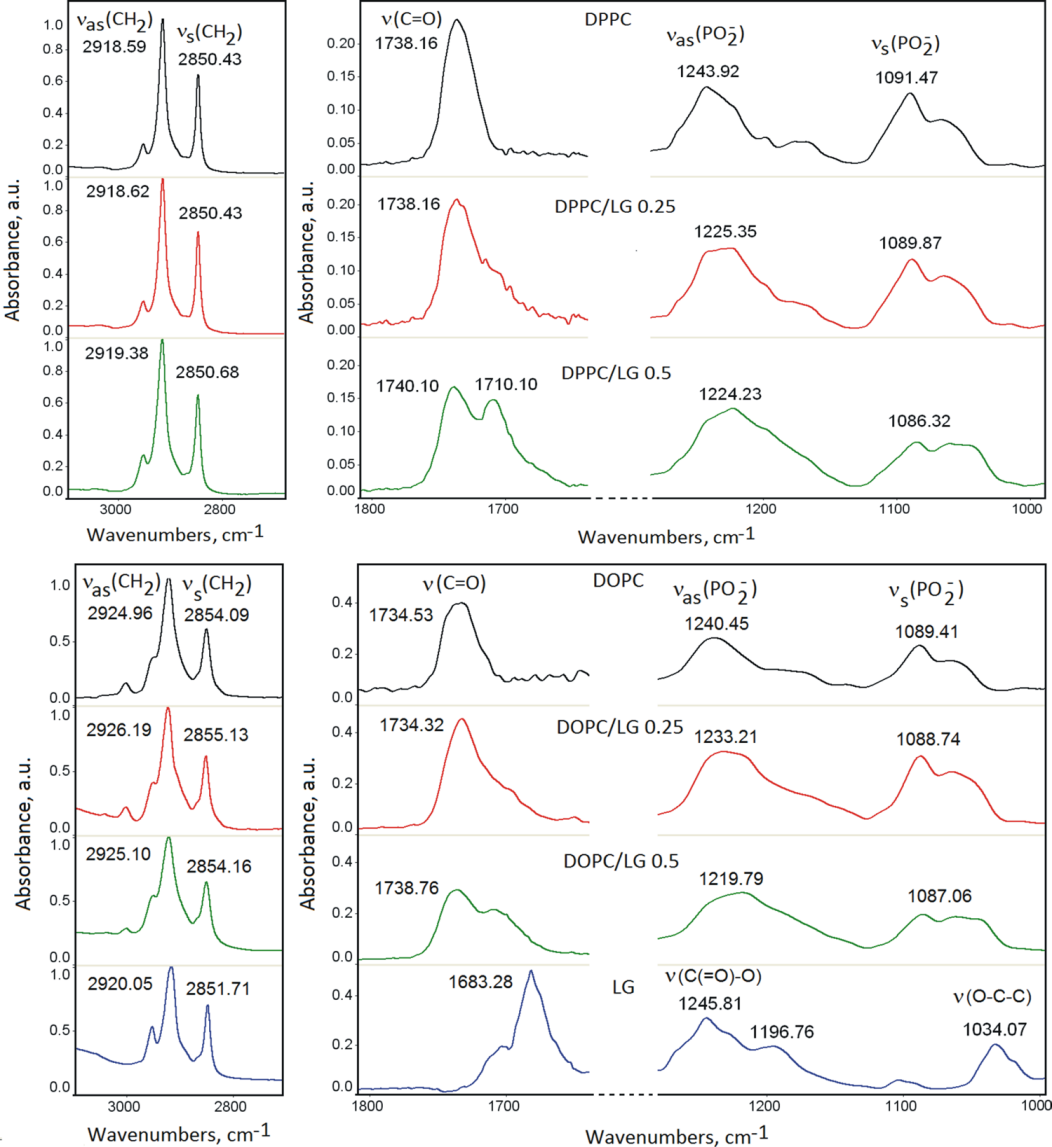
ATR-FTIR absorption spectra of the pure DPPC, DOPC or LG and mixed DPPC(DOPC)/LG (*x*_LG_ = 0.25 and 0.5) hydrated multibilayers

In Fig. 9 the ATR-FTIR absorption spectra of hydrated multibilayers consisting of DPPC or DOPC and different molar fractions of LG (0.25 and 0.5) can be seen. PC molecules possess several IR active groups. The high-frequency range (2920–2850 cm^−1^) of infrared spectra of PC membranes is represented by the bands of antisymmetric and symmetric stretching vibrations of CH_2_ groups (ν_as,s_(CH_2_)) of lipid hydrocarbon chains that form a hydrophobic core of the lipid bilayer. These bands are sensitive to the lateral packing of hydrocarbon chains. Spectral parameters (wavenumber of absorption maximum and bandwidth) of the ν_as,s_(CH_2_) bands monitor the alterations of the ratio of *trans* to *gauche* conformers of lipid CH_2_ moieties (Cieślik-Boczula et al. 2015). Comparing DPPC/LG with the DOPC/LG systems one can observe shift of the absorption maxima of both ν_as,s_(CH_2_) bands of DOPC/LG to a higher-frequency region and increase in the bandwidths of these two vibrational modes. This indicates that the DOPC/LG bilayers adopt a conformationally disordered *gauche*-rich fluid state while the DPPC/LG bilayers are enriched in the* trans* conformer population in the hydrocarbon part of the membrane. The absorption maxima of both ν_as,s_(CH_2_) bands for pure LG are between those of DPPC and DOPC, and simultaneously the peaks become broader/tighter and shifted to higher/lower frequencies than those of DPPC or DOPC, respectively. Such observation implies the intermediate ordering of hydrocarbon chains.

Several bands represent vibrations of the groups participating in the polar headgroup region of the membrane: antisymmetric N^+^–(CH_3_)_3_ stretching vibrations of choline, symmetric and antisymmetric stretching vibrations of PO_2_^−^ group, stretching vibrations of the C–O–P–O–C fragment, stretching vibrations of the ester carbonyl group C=O which represents the polar–apolar interface in the PC membrane (Cieślik-Boczula et al. 2015; Hereć et al. 2007). The C=O groups stretching band region between 1750–1700 cm^−1^ is one of the most intensive lipid polar group bands.

The changes of the C=O band position of hydrated pure PC multibilayers as a result of interactions with LG can be seen on the spectra. In the LG presence the contour of the C=O band broadens (*x*_LG_ = 0.25) and its maximum shifts to the higher-frequency region compared to the pure PC films, and at *x*_LG_ = 0.5 finally splits into two bands. This is due to contribution of the C=O stretching coming from the C=O ester groups of LG. In the spectrum of pure LG the band ascribed to the stretching vibrations of ester C=O group appears at 1683.28 cm^−1^. Its shift suggests participation of carbonyl groups in the interaction process. The other characteristics for the LG bands at 1245.81, 1196.76 and 1034.07 cm^−1^ correspond to the C(=O)–O stretching vibrations of ester groups, in-plane bending vibrations of the phenolic group and O–C–C stretching vibrations of the ester group, respectively (Fig. 9).

The asymmetric PO_2_^−^ vibrations ν_as_(PO_2_^−^) are very sensitive to hydrogen bonds formation (Cieślik-Boczula et al. 2015; Hereć et al. 2007). Therefore the frequency of antisymmetric stretching of PO_2_^−^ is a vibrational indicator of a level of lipid bilayer hydration and can be used to detect the H-bond interactions between the phosphate group of PC and the proton-donor groups of LG. For the PC multibilayers the position of this band is within the range of 1260 cm^−1^ (in the dry PC state) and 1220 cm^−1^ (in the fully hydrated PC state) (Ciesik et al. 2006). Insertion of LG molecules into the PC membrane leads to a shift to the lower frequency and broadening of the PO_2_^−^ asymmetric stretching mode. Such effect is indicative of a higher degree of hydration of the headgroups and can be readily interpreted in terms of hydrogen bonding to the PO_2_^−^ group, either LG or water molecules, influenced by the presence of the LG in the system. The band in the region between 1094 and 1085 cm^−1^ is associated with the symmetric stretching vibrations of the phosphate group ν_s_(PO_2_^−^) (Cieślik-Boczula et al. 2009). The shift to lower frequencies is also visible on the absorption spectra confirming the hydrogen bond connection between LG and the phosphate lipid part. Hence LG affects hydration near the PC headgroups, probably being positioned close to the phosphate and carbonyl groups of PC.

### TOF-SIMS Analysis

**Fig. 10:**
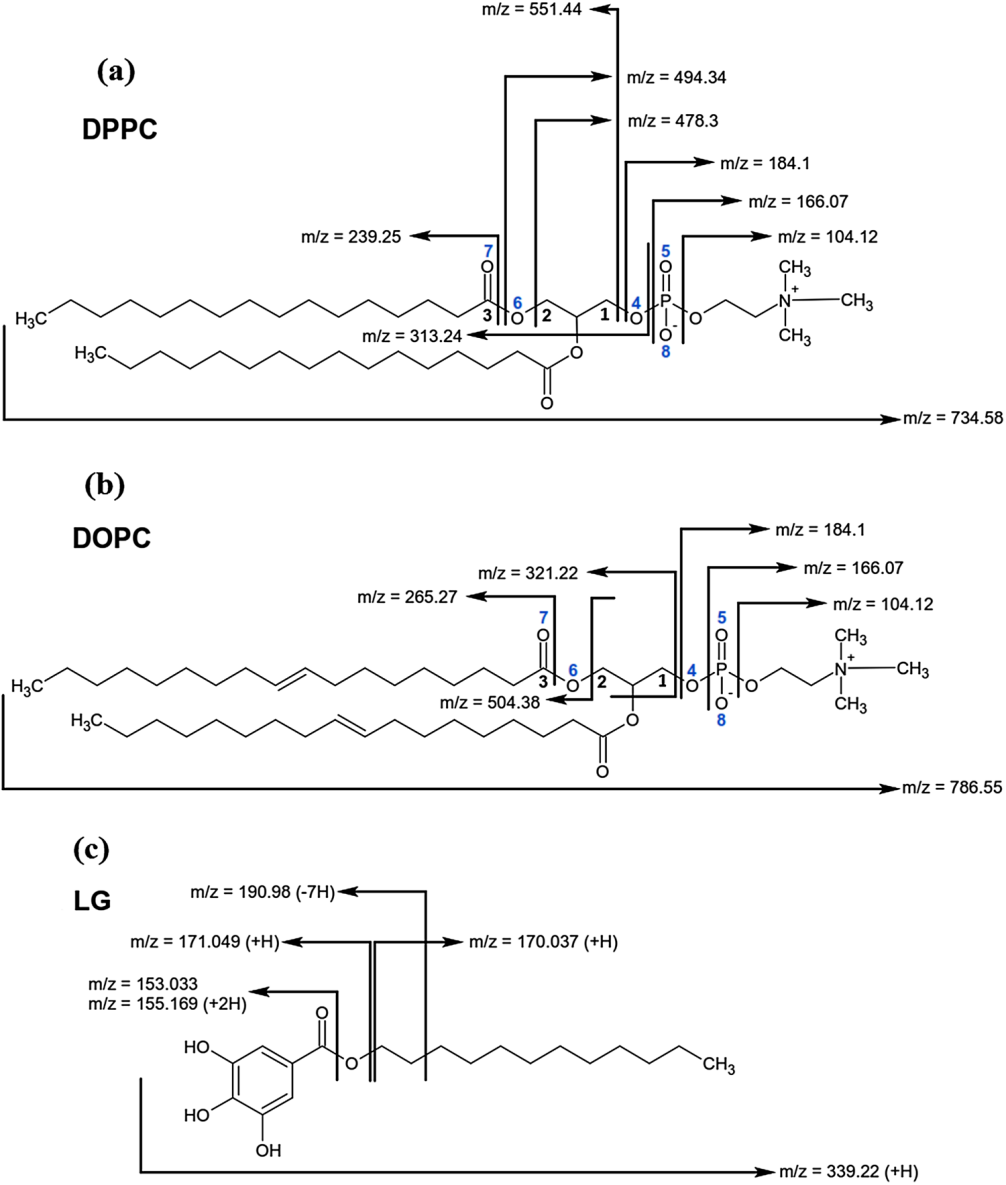
Structures and characteristic fragments of DPPC (**a**), DOPC (**b**) and LG (**c**)

To get better insight into the interactions between gallate moiety with the polar head groups of PC and to estimate mutual co-localization of these fragments of molecules in the monolayer, the TOF-SIMS analysis was conducted for the binary DPPC/LG and DOPC/LG monolayers at *x*_LG_ = 0.25 with regard to those of pure DPPC and DOPC, respectively. This technique allows chemical determination of compound distribution in the LB films based on the signal intensity of characteristic secondary ions (McQuaw et al. 2005). The molecular structures of DPPC, DOPC and LG are presented in Fig. 10 along with the marked binding cleavage site and the mass-to-charge ratio (m/z) of the main fragment ions.

No lateral heterogeneity was detected for both pure and binary monolayers proving that the monolayer structure was retained during the analysis. The increase of interaction strength between particular groups of both components makes the bond cleavage by the bombarding projectile more difficult. Simultaneously the bonds with neighbouring groups become weaker and therefore prone to rupture during primary ion bombardment. Hence the secondary ion yields of the formed fragments of PC are greatly enhanced as compared to a pure film.

**Fig. 11:**
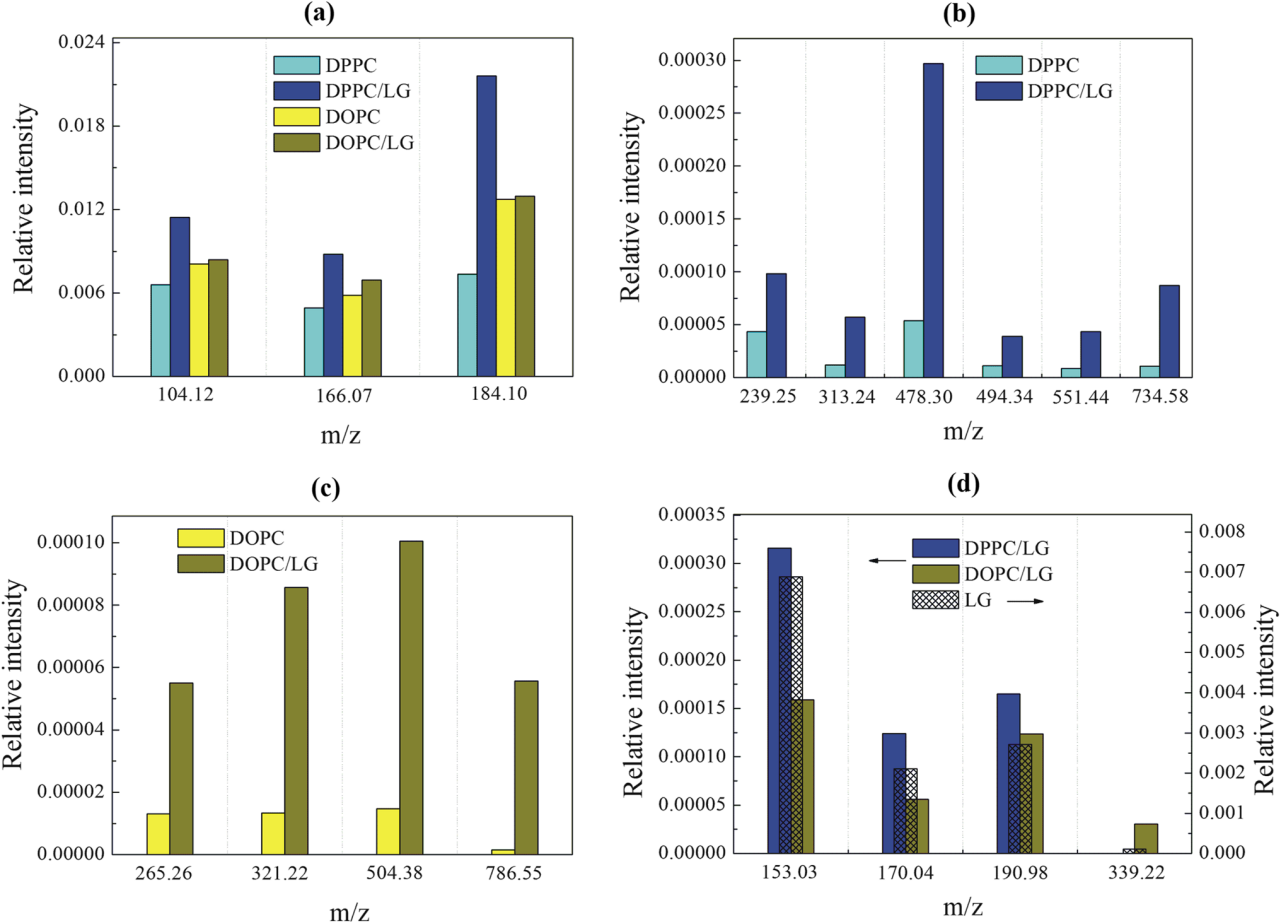
Relative intensity of the selected peak characteristic of: the PC head group for the DPPC, DPPC/LG, DOPC and DOPC/LG monolayers (**a**), the central region for the DPPC and DPPC/LG monolayers (**b**), the central region for the DOPC and DOPC/LG monolayers (**c**), the LG ions for the LG, DPPC/LG and DOPC/LG monolayers (**d**). The molecular ions of DPPC, m/z 734.58 (**b**), DOPC, m/z 786.55 (**c**) and LG, m/z 339.22 (**d**) are also inserted. (Color figure online)

Figure 11a shows the most abundant ions originating from the phosphocholine PC head. Peak at m/z 184.10 represents the (C_5_H_15_NPO_4_)^+^ ion. Other characteristic ions produced by PC head appear at m/z 104.12 ((C_5_H_14_NO)^+^) and m/z 166.07 ((C_5_H_13_PO_3_N)^+^) (Fig. 10a, b). As can be observed (Fig. 11a) intensity of all ions corresponding to the PC head of pure DOPC is slightly higher than that of DPPC. This is due to higher packing of DPPC molecules that reduces intensity yield of ions from the PC head. In the mid mass region (Fig. 11b) significantly higher intensity of pure DPPC ions at m/z 239.25 and 478.30 can be seen. This phenomenon is determined by intermolecular rearrangement that is accelerated by hydrogen transfer within the DPPC molecule (Murphy 1993). During the TOF-SIMS experiment after irradiation by the Bi_3_^+^ primary beam, hydrogen at C1 atom (Fig. 10a) is transferred through the C1–O4 bond, next O4–P and finally to P=O5 to retransform P=O5 into P–OH and P–O into P=O. In consequence, C1–O4 bond is broken. In the second step the other hydrogen is transported from the molecular environment into P–O8^−^, and as a result, the yield of positive phosphocholine ion with m/z 184.10 increases (Sostarecz et al. 2004). The same phenomenon is probably arranged symmetrically by hydrogen at C2 that hydrogen is transferred through the C2–O6 bond, next C3–O6 and finally into C3=O7, and then rearrangement of C3=O7 into C3–O7H and C3–O6 into C3=O6 takes place. In consequence, C2–O6 bond is broken releasing an ion with m/z 478.3 of higher intensity (Fig. 10a). Then the other hydrogen is transferred from the molecular environment into C3–O7H leading to C3–O7H_2_^+^, and as a result, the C3–O6 bond in this group is broken releasing the ion of m/z 239.25. In the case of pure DOPC a similar behaviour of ions of m/z 265.26 and 504.38 (Fig. 10b) is observed. These correspond to the ions of pure DPPC at m/z 239.25 and 478.30, respectively. Similar changes of signal intensity of ions at m/z 265.26 and 504.38 arise from the higher intensity of ion at m/z 184.10 formed by breaking the C1–O4 bond. The molecular ions of DPPC and DOPC occur at m/z 734.58 and 786.55, respectively (Fig. 11b, c). Higher intensity of the molecular ion is provided by the DPPC monolayer due to its ordered and tightly packed structure which improves efficiency of molecular ion desorption. Emission of molecular ions and ion clusters away from the impact zone was also reported for the highly packed self-assembled monolayer (Graham and Ratner 2002).

**Fig. 12:**
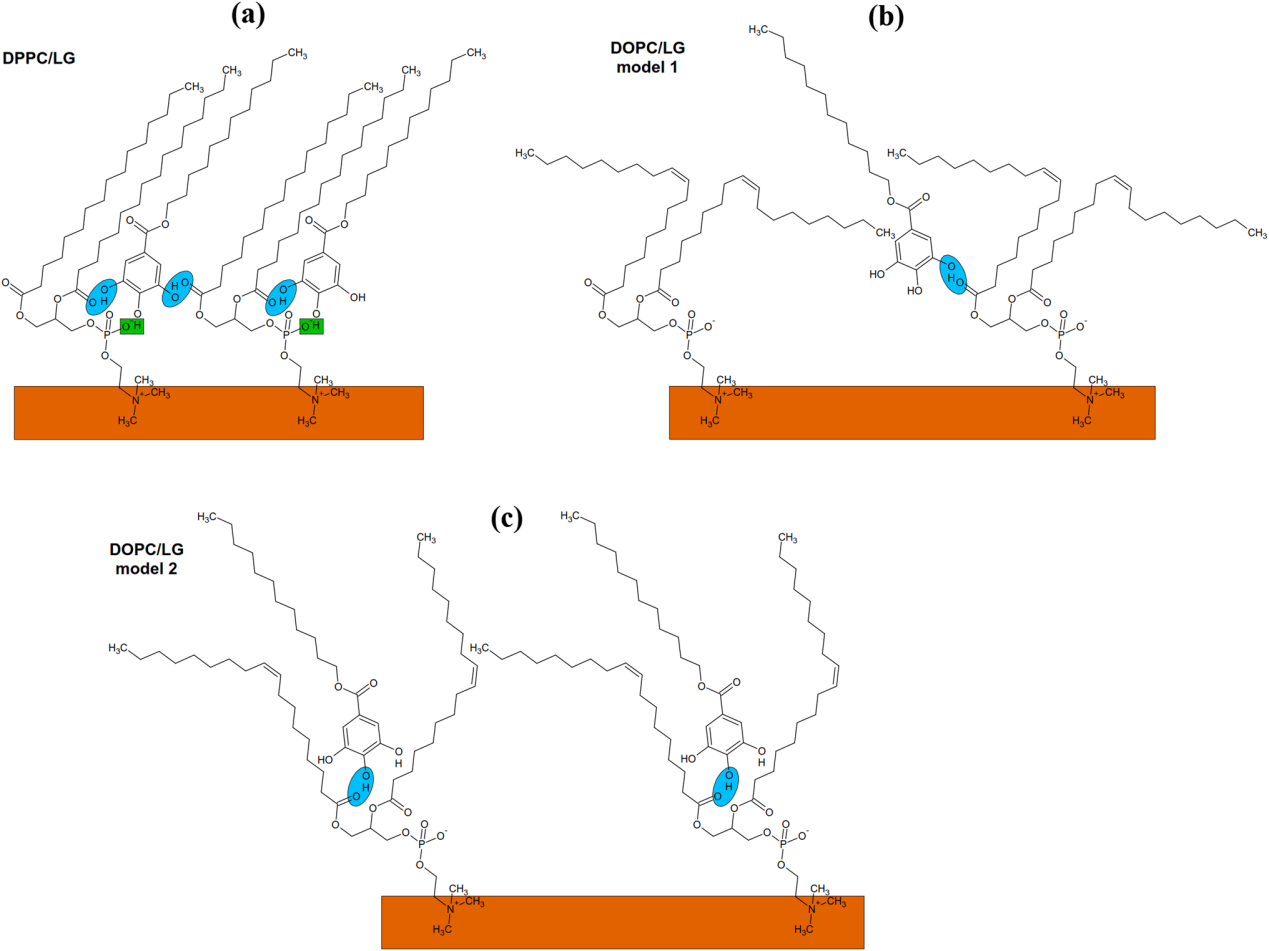
Models illustrating the interactions between the LG and DPPC molecules (**a**) and the LG and DOPC molecules (**b, c**) along with their localization with respect to each other in the monolayer

To illustrate how insertion of LG into the DPPC or DOPC monolayer affects the molecular organization, simple models are presented in Fig. 12. As shown in Fig. 11a in the presence of LG the signal intensity of PC ions at m/z 104.12, 166.07 and 184.10 increases 1.74, 1.78 and 2.94 times, respectively. It proves unambiguously the direct donor–acceptor interactions between the LG and DPPC molecules provided by hydrogen bonds between hydroxyl groups of LG (marked blue in Fig. 12a) and hydrogen transfer from one hydroxyl group to phosphate group (marked green) which increases the yield of 184.10 ion as it was described above. For the DOPC/LG system two models (1 and 2) can be proposed (Fig. 12b, c, respectively). In model 1 (Fig. 12b) the galloyl group of LG molecule is localized at the first carbonyl group of PC (blue) linked by hydrogen bond. In that way there is no direct access to the phosphocholine group and therefore the signal intensities of phosphocholine ions coming from the DOPC/LG monolayer are similar to those of pure DOPC due to the lack of hydrogen transfer between the hydroxyl group and phosphate groups. In the second model (model 2, Fig. 12c) the left CH chain is a little bit straightened and twisted around the carbonyl group that is involved in the hydrogen bond whereas the right chain is slightly straightened. In this rearrangement LG can go deeper between the two CH chains but still direct access to the phosphocholine head is not possible. Model 2 seems to be more realistic but two scenarios should be considered in the real world. In the mid mass range the signal intensity of ion at m/z 478.30 is 5.5 times higher in comparison to that of pure DPPC. This is strongly correlated with the increased 184.10 ion yield due to hydrogen transfer between the hydroxyl group of LG and DPPC. There are also higher intensities for all other ions at m/z 239.25 (2.26× higher than for pure DPPC), m/z 313.24 (4.74×), m/z 494.34 (3.50×) and m/z 551.44 (5.02×). For each case a different increased yield is determined by coupling with the intensity yield of complementary ions, e.g. 166.07 and 313.24, 184.10 and 551.44 as it is shown in Fig. 10a. For DOPC intensity of the mid mass range ions increases monotonically which indicates that the ionization mechanism is not combined with the phosphocholine head but it is caused mainly by orientation of the CH chains towards the primary Bi_3_^+^ beam and different packing of LG within the DOPC monolayer.

Interestingly, intensity of molecular ion of DOPC after the addition of LG is 38.3 times higher than that of pure DOPC. This is due to weak binding of LG into DOPC through the hydrogen bond that is favourable for desorption of molecular species. In the presence of LG the yield of DPPC molecular ion increases only eight times indicating that molecules are more strongly bound within the DPPC/LG monolayer due to the additional interactions by hydrogen transfer between the hydroxyl group of LG and the phosphate group of DPPC. As a result, in the molecular desorption region (far away from the impact region) (Graham and Ratner 2002) desorption of molecular ions of DPPC is more difficult than that of DOPC which is involved in the interactions with LG only by the hydrogen bond.

Figure 11d shows intensity of characteristic ions for LG. One can observe that the yield of ions at m/z 153.03, 170.04 and 190.98 decreases about twice more slowly for DPPC/LG than it is observed for DOPC/LG. The reason for that can be found in the molecular arrangement of the DPPC/LG and DOPC/LG monolayers. Additionally, the molecular ion of LG is not observed in the DPPC/LG system while it gives a very intensive signal for DOPC/LG. It is likely that LG linked strongly with DPPC by the hydroxyl group owing to the hydrogen donor–acceptor mechanism is not desorbed whereas in the DOPC/LG monolayer LG is linked only by the weak hydrogen bond which favours desorption of a large number of LG molecular ions in the molecular desorption region.

## Discussion

In the paper the model membranes made of DPPC and DOPC were used to study the effect of Chol or LG on the phospholipid monolayer miscibility, physical state and ordering as well as overall stability. In the absence of cholesterol, PLs form two-phase lipid regions, liquid condensed and liquid expanded that are rich in the high melting temperature DPPC and the low melting temperature DOPC, respectively. Contrary to DPPC, the DOPC molecule contains unsaturated hydrocarbon chains which cannot occur in the all-*trans* conformation. Therefore DOPC exists in the liquid–crystalline (fluid) phase at the experimental temperature. Consequently, because of the presence of C18 chains with one double bond which induces a kink, the DOPC monolayer is less condensed (Fig. 4) and its thickness is lower than that estimated for DPPC (Fig. 5). The mixed DPPC/DOPC monolayer exhibits the thickness intermediate between those of pure components. The reason for that can be found in partial miscibility of DPPC and DOPC revealed in the formation of separated domains of one phase in another phase as shown by means of BAM (Fig. 2). Domain formation in the DPPC/DOPC system is associated with the decrease in the monolayer condensation as well as ordering (lower $C_{{\text{s}}}^{{ - 1}}$) (Fig. 4) and weakening of attractive interactions expressed by the positive values of *A*_exc_ (1.1 Å^2^/molecule) and ∆*G*_exc_ (1277.8 J/mol) (Figs. 6, 7, respectively).

Cholesterol with an equimolar mixture of DPPC and DOPC forms the micron-scale liquid domains visible in the BAM images (Figs. 2, 3). Their thickness successively increases with the Chol mole fraction. The observed coexisting liquid phases are in agreement with the phase diagrams of the ternary DPPC/DOPC/Chol system reported in the literature (Veatch and Keller 2002, 2003; Veatch et al. 2004; Almeida 2009). According to McConnell and Radhakrishnan, the equivalence point of the condensed complexes formation is between 25 and 50% of Chol (McConnell and Radhakrishnan 2003). The solubility limit of cholesterol in the DOPC multilayers is 40 mol% (Hung et al. 2007). The condensing effect for the DPPC-rich liquid-ordered *L*_o_ phase is stronger than for the DOPC-rich liquid-disordered *L*_d_ phase (McConnell and Radhakrishnan 2003; Ma et al. 2016). Ma et al. proved that Chol accumulates in a larger amount in the DPPC-stabilizing *L*_o_ phase within the *L*_d_ matrix phase. Cholesterol increases the electron density in the lipid-chain region in the DPPC-rich phase more than in the DOPC-rich phase (Ma et al. 2016). For equal mole fractions of DPPC and DOPC and 20% Chol at 25 °C, partition of Chol into the *L*_o_ and *L*_d_ phases is 31% and 10–11%, respectively. As an effect Chol increases the monolayer thickness of the *L*_d_ DOPC phase while it decreases that of the *L*_o_ DPPC phase owing to matching the hydrophobic parts of PL and cholesterol. Hence the successive increase in the average domain thickness with the Chol mole fraction is observed (Fig. 5). After Chol complexing its excess simply mixes with the complexes. Hence the monolayer of 1:1 DOPC/DPPC+75% of Chol is in the one uniform liquid phase at 20 °C seen as the homogeneous monolayer in the BAM image (Fig. 2). Cholesterol ordering effect ($C_{{\text{s}}}^{{ - 1}}$ increase) is driven by the steric factors which lead to favourable interactions revealed in the negative values of excess area, excess and total Gibbs energy of mixing (Figs. 6, 7, 8).

LG analogously to Chol can modulate the fluidity and ordering of the membrane leaflet, thus influencing its function. LG forms the disordered monolayer at the air–water interface similarly to DOPC (Fig. 4). Although pure DPPC monolayer exists as the liquid-condensed ordered phase, its mixture with DOPC gives DPPC/DOPC monolayers of elasticity close to that of DOPC and LG. The boundary factor to induce the fluidizing or solidifying effects is related deeply to a mixture composition and stoichiometry. It was previously reported (Jurak and Miñones 2016) that the interactions between DPPC and LG lead to increased order in the packing of molecules when the amount of LG was 25%. This was attributed to the change of the PC molecules orientation in the Langmuir monolayer which first promotes the nucleation of LC domains, and finally the increase of the monolayer stiffness (Jurak and Miñones 2016). At higher concentration of LG the DPPC membrane tends to fluidize. Unsaturated DOPC makes very compact assembling difficult. In consequence, in the DPPC/DOPC/LG system at *x*_LG_ = 0.25 the phase behaviour is almost the same as for the DPPC/DOPC monolayer but the mean area per molecule in the ternary system decreases by about 9.8 Å^2^. With the further addition of LG (*x*_LG_ = 0.5), the border of ordered domains blurs which is associated with the fluidizing effect of LG due to disturbing the ordered structure of DPPC-enriched domains. This strongly supports the increased mutual miscibility between DPPC, DOPC and LG in the monolayer state. Hence similarity in the phase behaviour of LG and DPPC/DOPC maintain the membrane fluidity.

Accommodation of Chol or LG within the phospholipid monolayer is accompanied by the decreasing molecular area which, excluding *x* = 0.25, is lower in the DPPC/DOPC/LG system as the LG area is lower than that of Chol at that surface pressure. However, in this system the thickness and packing of monolayers change only little with the increasing LG due to the similar phase state/condensation degree of the LG and DPPC/DOPC monolayer (Figs. 4, 5). Takai et al. found that the alkyl gallates are more stable in the liquid phase of DPPC/DOPC than in the solid phase and their stability is mainly determined by the membrane components (Takai et al. 2011). Moreover, the partition constant for LG to DOPC is twice higher than that to DPPC. Its value for the 1:1 DPPC/DOPC mixture is intermediate and increases with the increasing ratio of DOPC to DPPC (Takai et al. 2011). As reported previously (Jurak and Miñones 2016) better miscibility and favourable composition were revealed for the mixed monolayers of LG with the unsaturated phospholipid DOPC. As the DOPC monolayer is less condensed than that of DPPC and has more intermolecular space, the LG matching is better. The attractive interactions of LG with DPPC are weaker if the LG mole fraction is > 0.25. The BAM images indicate partial miscibility in DPPC/DOPC/LG leading to phase separation or domain formation depending on the surface pressure. The major driving force for these processes are the very unfavourable interactions between DPPC and DOPC. However, incorporation of LG to the DPPC and DOPC mixture, similarly to Chol, reduces the repulsive interactions converting them into being more attractive, thus increasing the ternary system stability (Fig. 8). The strength of interactions is higher in the systems with Chol. But contrary to Chol, LG is considered to localize preferentially in the regions enriched in unsaturated PC in which serves its antioxidant function. More negative values of Δ*G*_exc_ and Δ*G*_mix_ indicate stronger attractive interactions. Hence the most favourable composition of ternary systems was that at *x*_Chol or LG_ = 0.5. In spite of smaller value of critical packing parameter and thus different shape of molecule than that of Chol, similarly to the role of Chol, LG can stabilize the membrane domain structure. However, the mechanism of stabilization can be quite different. Cholesterol exerts a compacting effect on PC. It induces conformational ordering of the hydrocarbon chains and thickens the membrane (Marquardt et al. 2016). Modifying the PC headgroup interactions LG plays a significant role in modulating the membrane organization. The fact that PC and LG form intermolecular hydrogen bonding contributes to the increasing stability of mixed layers.

To understand this mechanism better it should be taken into account that the PC headgroups with three methyl groups do not allow for interheadgroup hydrogen bonding and their orientation is parallel to the air–water interface (Miñones et al. 2002). Moreover, the area requirement of the large hydrated head group of the DPPC molecule prevents the erection of the aliphatic chains on compression. Hence, the chains of DPPC molecules in the liquid-condensed phase are tilted by about 30° to compensate for the head–tail mismatch (Ma and Allen 2006). On the other hand, the phenolic hydroxyl groups of LG serving as the hydrogen bond donors can be involved in the interactions with oxygen atoms on the PL as the hydrogen bond acceptors favouring the miscibility. As indicated by Sirk et al. the presence of the gallate moiety and its *cis* configuration with ring B facilitate the formation of multiple hydrogen bonds with lipid headgroups (Sirk et al. 2008). Accordingly, polyphenols with the gallate moiety formed 40% more hydrogen bonds than those without it. Therefore, it is likely that the hydroxyl groups of galloyl moiety of LG can form hydrogen bonds with the oxygen atoms in the phosphate and the glycerol–fatty acid ester groups of PC headgroups while its lauryl tail resides among the phospholipid acyl chains. In consequence, the polar heads of PC oriented first almost parallel to the air–water interface (Miñones et al. 2002) change the orientation (tilting) due to the various LG–PC headgroup interactions. The approximation of polar heads owing to hydrogen bonding yields apparent condensation of the monolayer evidenced by smaller areas per molecule consistent with the *π*–*A* isotherm (Fig. 1c). However, LG keeps the DPPC/DOPC membrane fluidity which can result from either bending or tilting of the PC chains. The similar behaviour was observed for the phosphatidylethanolamine (DPPE)/Chol monolayer (McQuaw et al. 2005) for which the increase in molecular density (smaller area per molecule) upon the addition of cholesterol was accompanied by the DPPE membrane fluidization contrary to DPPC.

Moreover, LG being an antioxidant molecule is able to protect the unsaturated lipids from oxidation by free radical scavenging. The studies of behaviour of α-tocopherol (Marquardt et al. 2013), which similarly to LG is an antioxidant, with respect to the lipids reported by Marquardt et al. show that location of α-tocopherol within the membrane correlates precisely with its antioxidant activity (Marquardt et al. 2013). The behaviour of LG seems to be similar to that of TF. Both Chol and LG can be a spacer to fill the free space between phospholipid molecules. The –OH group of Chol is localized in proximity of the –C=O group of phospholipid (Chen and Tripp 2008). The spectroscopic analysis of the hydrated bilayers proves that the localization of galloyl group takes place also in the polar membrane zone. The shift of the peak corresponding to the asymmetric stretching vibrations of phosphate PO_2_^−^ groups of the polar headgroup region can be associated with possible rearrangement of PO_2_^−^ groups owing to their interactions with LG. Additionally, the changes of signal intensity of characteristic ions obtained by TOF-SIMS (Fig. 11) point out the mutual interactions between the headgroups of both components. By analogy to α-tocopherol (Marquardt et al. 2013), it is likely that LG antioxidant activity is revealed solely at the membrane surface, not within the hydrocarbon matrix. LG does not rather penetrate the hydrocarbon matrix but it is positioned at the apolar–polar (lipid–water) interface which ensures more effective inhibition of lipid oxidation. However, some differences in LG depth within the PC molecules can be imposed by the kind of hydrocarbon chains. The TOF-SIMS analysis confirmed that LG is deeply submerged within DOPC, closer to the double bonds which correlates with its antioxidant activity.

The interactions of lipophilic antioxidants with PC could be a relevant mechanism in the protection of membrane against oxidation (Oteiza et al. 2005). They determine changes in the membrane physical properties and the rates of membrane lipid oxidation. Participating in the hydrogen bonds with the polar head groups at the lipid–water interface LG could reduce the access of external and internal aggressors (i.e. oxidants) to the membrane thus preserving its structure and function. The antioxidant activity of LG seems to be primarily due to the gallate moiety but the hydrophobic dodecyl tail could also play a role in modulation of membrane physical properties (Kubo et al. 2002). Hence, the physical state (condensed or fluid) adopted by the PL in the mixed monolayers and the hydrophobic mismatch between the components drive mainly the organization of the molecules at the interface.

## Conclusions

The phase behaviour, thickness and miscibility of monolayers with the equal mole fractions of DPPC and DOPC and steadily increasing LG concentration were considered compared to the systems with cholesterol (Chol). Chol is primarily a structural component of biological membranes. LG can interact with the lipid components of membrane and simultaneously being an antioxidant molecule, it is able to protect the unsaturated lipids from oxidation by free radical scavenging. Chol in the DPPC/DOPC monolayers causes their condensation leading to an increase in density of lipid molecules and their ordering, thus improving the film stability. LG practically retains the DPPC/DOPC membrane fluidity and minimizes the unfavourable interactions between DPPC and DOPC also increasing the phospholipid miscibility and stability. As regards the distribution between the coexisting phases, LG preferentially localizes in more fluid areas, which is imposed by antioxidant activity, whereas Chol in more ordered ones. This results from differentiated affinity of both molecules for unsaturated or saturated PL. The favourable interactions contribute to the formation of heterogeneities within the membrane. They seem to reflect the specialization of various regions in the membrane processes.
